# Autopsy in the President’s Case

**Published:** 1881-09-20

**Authors:** 


### *AUTOPSY IN THE PRESIDENT'S CASE.*

The death of President Garfield occurred at 10.35 p.m., September 19th 1881. The following account of the autopsy was published:

The following official bulletin was prepared by the surgeons who were in attendance on the late President:

By previous arrangement a post-mortem examination of the body of President Garfield was made in the presence and with the assistance of Drs. Hamilton, Agnew, Bliss, Barnes, Woodward, Reyburn, Andrew Smith, of Elberon, and Adjutant-Assistant Surgeon D. S. Lamb, of the Army Medical Museum, of Washington. The operation was performed by D. S. Lamb. It was found that the ball, after fracturing the right eleventh rib, had passed through the spinal column in front of the spinal canal, fracturing the body of the first lumbar vertebræ, driving a number of small fragments of bone into the adjacent soft parts, and lodging below the pancreas, about two and a half inches to the left of the spine, behind the peritoneum, where it had become completely encysted. The immediate cause of death was secondary hemorrhage from one of the mesenteric arteries adjoining the track of the ball, the blood rupturing the peritoneum and nearly a pint escaping into the abdominal cavity. This hemorrhage is believed to have been the cause of the severe pain in the lower portion of the chest complained of just before death. An abscess cavity, six inches by four in dimensions, was found in the vicinity of the gall-bladder, between the liver and the transverse colon, which was strongly adherent. It did not involve the substance of the liver, and no communication was formed between it and the wound. A long suppurating channel extended from the external wound between the loin muscles and the right kidney almost to the right of the groin. This channel, now known to be due to the burrowing of pus from the wound, was supposed, during life, to have been the track of the ball. On an examination of the organs of the chest, evidence of severe bronchitis were found on both sides of the bronchi, though to a much less extent of the left. The lungs contained no abscesses, and the heart no clots. The liver was enlarged and fatty, but free from abscesses, nor were any found in any other organ except the left kidney which contained near its surface a small abscess about ½ of an inch in diameter.

In reviewing the history of the case in connection with the autopsy, it is quite evident that the different suppurating surfaces, and especially the fractured spongy tissue of the vertebræ, furnish sufficient explanation of the septic condition which existed.

We have heretofore refrained from commenting upon the case of the President, feeling that the physicians in attendance were intelligent and competent men, and were doing all that could be done. We regard the criticisms as to the treatment, made by the newspaper writers, and especially by medical writers, as uncalled-for and in bad taste. The autopsy, it is true, discloses that on some points of diagnosis of the medical men was at fault, particularly in regard to the track and locality of the ball, but the error made was one that would have been made perhaps in a thousaud similar instances by any number of surgeons, and did not in any serious way change the final result, which was inevitable under any treatment that might have been adopted.

